# A descriptive multilevel analysis associating COVID-19 with polymyositis: from genetic markers and candidate mediators to clinical hematological profiles

**DOI:** 10.3389/fmed.2026.1775960

**Published:** 2026-04-09

**Authors:** Miaomiao Chen, Hongmei Li, Xia Tong, Xin Zhang

**Affiliations:** 1 Department of Respiratory and Critical Care Medicine, Tianjin Chest Hospital, Tianjin, China; 2 Department of Neurology, Affiliated Hospital of Traditional Chinese Medicine of Southwest Medical University, Luzhou, China; 3 Department of Gastroenterology, West China (Airport) Hospital of Sichuan University, Chengdu, China

**Keywords:** clinical retrospective analysis, COVID-19, mediation analysis, Mendelian randomization, polymyositis

## Abstract

**Objectives:**

Observational studies suggest an association between Coronavirus Disease 2019 (COVID-19) and polymyositis (PM), but causal inference s limited by confounding. This study adopted a multilevel exploratory framework to investigate potential relationships. We used two-sample Mendelian randomization (MR) to assess the causal effect of severe COVID-19 on PM, multi-omic analyses to screen for potential mediators, and retrospectively compared hematological profiles between severe and non-sever COVID-19 cases.

**Design:**

A two-sample MR with mediation analysis and a single-center retrospective cohort analysis.

**Setting and participants:**

Genetic instruments for severe COVID-19 (exposure), PM (outcome) and candidate multi-omic mediators (91 inflammatory proteins, 4,907 circulating plasma proteins, 731 immune-cell traits, and 1,400 plasma metabolites) were obtained from genome-wide association studies (GWAS). The clinical study included 108 hospitalized patients with PCR-confirmed COVID-19, classified into severe and non-severe subgroups.

**Main outcomes:**

The main outcome in the MR analysis was the causal odds ratio (OR) of PM per genetically predicted increase in the risk of severe COVID-19. Secondary outcomes included the identification of mediating biomarkers and differences in hematological indices between severe and non-severe COVID-19 patients.

**Results:**

MR analysis suggested a potential causal effect of severe COVID-19 on PM (IVW OR = 1.65, 95% CI: 1.36–2.01, *p* < 0.01) with no reverse causality. Mediation analysis highlighted several candidate mediators (KIAA1024, RNASE1, EGFLAM, CAPZA1, NRG3, IL31). Functional enrichment implicated ErbB signaling and “Overview of proinflammatory and profibrotic mediators pathway.” The clinical analysis showed that severe COVID-19 patients had altered neutrophil, lymphocyte, and platelet counts, and elevated levels of muscle enzymes and IgE. After age and sex adjustment, lymphocyte count, CK-MB, LDH, and HBDH remained independently associated with severe COVID-19 (all adjusted *p* < 0.05).

**Conclusion:**

This multilevel exploratory study provides preliminary genetic evidence suggesting a potential association between severe COVID-19 and polymyositis, along with candidate molecular mediators. The clinical analysis revealed distinct hematological profiles in patients with severe COVID-19. These findings should be interpreted as preliminary clues rather than definitive mechanistic evidence. Future studies with independent validation cohorts, larger sample sizes, and longitudinal follow-up for incident myositis are needed to confirm these observations.

## Introduction

The global burden of the Coronavirus Disease 2019 (COVID-19) pandemic extends beyond acute respiratory illness to include a spectrum of post-infection complications, among which autoimmune sequelae are of growing concern (1, 2). Specifically, polymyositis (PM), a rare and debilitating idiopathic inflammatory myopathy, characterized by chronic muscle inflammation and weakness, has been reported in association with COVID-19, though the nature of this link remains unclear (3, 4).

A plausible biological pathway links COVID-19 to autoimmune pathogenesis. Infected individuals exhibit significant immune dysregulation, including elevated levels of key inflammatory cytokines (e.g., IL-6, IL-12, IFN-*γ*) (5, 6), as well as broader metabolic abnormalities and altered plasma protein profiles (7–9). These perturbations are also implicated in the pathogenesis of PM, suggesting a potential mechanistic overlap. Furthermore, cases of viral myositis (a general term for muscle inflammation induced by viral infection, distinct from the specific diagnosis of PM) following COVID-19, potentially due to direct viral invasion or immune-mediated damage, have been documented (10). However, established observational associations between COVID-19 and PM are susceptible to confounding and reverse causality, precluding definitive causal inference.

To address this uncertainty and explore the potential causal link, we employed Mendelian randomization (MR), a genetic epidemiological method that uses genetic variants as instrumental variables (IVs) to assess potential relationships while minimizing confounding. Applying a two-sample MR design, we first aimed to investigate whether genetic predisposition to severe COVID-19 is associated with an increased risk of PM.

Beyond assessing the genetic association, exploring the potential mediating pathways is critical. The specific biological layers—potentially involving inflammatory proteins, immune cell dysregulation, metabolites, and circulating plasma proteins—that might be associated with this relationship remain largely uncharacterized. Therefore, our second objective was to implement an exploratory multi-omic MR framework to systematically screen for candidate mediating biomarkers across these diverse biological levels.

Finally, we performed an independent, descriptive retrospective analysis of hematological and muscle enzyme profiles in severe versus non-severe COVID-19 patients. This clinical component was explicitly designed as an independent descriptive analysis; it was not intended to test the hypothesis derived from MR, nor to measure the candidate mediators identified by MR, but to delineate the real-world phenotypic landscape of immune dysregulation associated with severe COVID-19. By characterizing these clinical abnormalities, we aimed to provide biological context that supports the phenotypic plausibility of the genetically identified candidate mediators (e.g., KIAA1024, RNASE1, EGFLAM, CAPZA1, NRG3, IL31), thereby improving the interpretability of our multi-omic findings.

Thus, this study was designed to explore the severe COVID-19-PM link through a multilevel, exploratory approach by assessing evidence for a causal relationship using MR, identifying candidate mediators across multi-omic domains, and providing a parallel clinical description of hematological profiles in COVID-19 patients to generate preliminary integrative hypotheses.

## Materials and methods

### Study design

**Figure 1:**
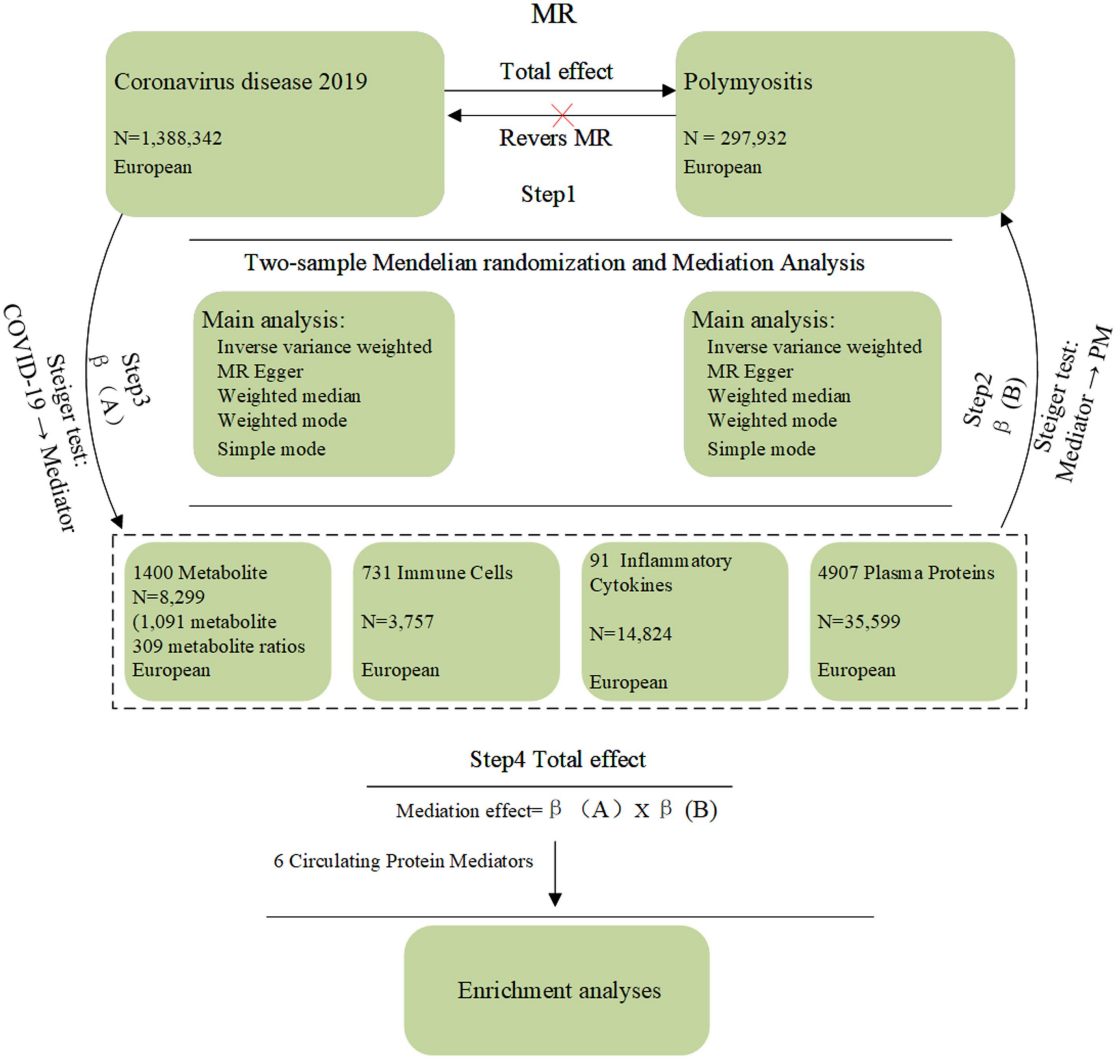
Flowchart of mediated MR analysis. This study employed a two-sample MR design to investigate the potential association between Coronavirus Disease 2019 (COVID-19) and polymyositis (PM), followed by an exploratory multi-omic mediation analysis. Step 1: Primary MR analysis was performed to estimate the total effect of COVID-19 on PM, with reverse MR conducted to assess potential reverse causality. Step 2: MR was used to estimate the effect of each potential mediator on PM (Path B). Step 3: MR was used to estimate the effect of COVID-19 on each potential mediator (Path A). Step 4: Mediation effects were calculated as the product of Path A and Path B coefficients, and significant mediators were identified. Prior to mediation analysis, Steiger directionality tests were performed to confirm the causal direction of all paths (COVID-19 to mediators and mediators to polymyositis). Finally, functional enrichment analyses were conducted on the identified candidate mediators to explore relevant biological pathways.

This study integrated a two-sample MR design (to explore potential associations and screen for candidate mediators) with a clinical retrospective cohort analysis (to characterize severe COVID-19 phenotypes). Figure 1 outlines the workflow of the mediated MR analysis for the severe COVID-19-PM association.

### Data sources for Mendelian randomization

We utilized publicly available summary-level GWAS data with European ancestry (consistent across exposure, outcome, and mediators to avoid population stratification) from the following resources:

Severe COVID-19 (exposure): Genetic instruments for severe COVID-19 were obtained from the COVID-19 Host Genetics Initiative (release 5, GWAS ID: GCST011075), comprising 5,101 cases and 1,383,241 controls of European ancestry (11).

Polymyositis (PM, outcome): Summary statistics for PM were sourced from the FinnGen consortium (release R12), which included 484,547 participants (287 cases and 484,260 controls).

Potential Mediators: To identify mediating biomarkers, we incorporated exploratory GWAS data for:

Ninety-one inflammatory proteins from the Olink Targeted Inflammation Panel (14,824 individuals) (12).

A total of 731 immunocyte phenotypes from a study of 3,757 Sardinians, including absolute cell counts, relative counts, and median fluorescence intensities (13).

A total of 1,400 plasma metabolites (including 1,091 metabolites and 309 ratios) from a metabolomics study of 8,299 samples (14).

A total of 4,907 circulating plasma proteins from a large-scale proteomics GWAS of 35,559 individuals of European ancestry (15).

### Selection of genetic instruments

The selection of IVs was rigorously performed to satisfy the key assumptions of MR (relevance, independence, no horizontal pleiotropy). GWAS harmonization was first conducted using the TwoSampleMR R package (v0.5.6): all genetic variants were aligned to the forward strand, and allele frequencies were matched between exposure, outcome, and mediator datasets. For all exposure–outcome pairs (COVID-19–PM, COVID-19–mediators, and mediators–PM), palindromic SNPs were harmonized using the harmonise_data function from the TwoSampleMR package with the default parameter action = 2. This approach infers strand orientation based on effect allele frequencies (EAF); SNPs with ambiguous orientation were excluded (mr_keep = FALSE). All GWAS used in this study provided EAF, enabling this strand-inference procedure. After harmonization, 16 SNPs were retained for the primary MR analysis. Subsequently, one SNP (rs2597569) was identified as a horizontal pleiotropy outlier by the MR-PRESSO test and was excluded, leaving 15 SNPs for the primary MR analysis (Supplementary Table S1).

SNPs were selected using phenotype-specific relaxed significance thresholds according to the availability of genome-wide significant loci, with a default threshold of *p* < 5 × 10^−8^ for each exposure trait and a relaxed threshold of *p* < 1 × 10^−5^ when genome-wide significant loci were insufficient. These SNPs were clumped to ensure independence (linkage disequilibrium threshold *r*^2^ < 0.001 within a 10,000 kb window) using the 1,000 Genomes Project (European ancestry) as the reference panel. To minimize horizontal pleiotropy, we excluded any SNPs that were directly associated with the outcome (PM) at a significance level of *p* < 0.05. The F-statistic [F = R^2^ × (n-2)/(1-R^2^)] was used to evaluate instrument strength, and only IVs with F-statistic > 10 were included. The basic characteristics and F-statistic values of the final IVs for severe COVID-19, PM and all potential mediators are listed in Supplementary material.

### Mendelian randomization and sensitivity analyses

The primary method for causal estimation was the inverse-variance weighted (IVW) method. Robustness of the findings was assessed using supplementary methods, including weighted median, MR-Egger, and weighted mode. Sensitivity analyses included Cochran’s Q test for heterogeneity, MR-Egger intercept and MR-PRESSO global tests for horizontal pleiotropy, and leave-one-out analysis for individual variant influence. All analyses followed STROBE-MR guidelines.

### Mediation analysis

A two-step MR approach was employed to investigate exploratory mediation. Prior to mediation analysis, Steiger directionality tests were performed to confirm the direction of causality between the exposure (severe COVID-19) and mediators, as well as between mediators and the outcome (PM), ensuring that the inferred causal directions were consistent with our research hypothesis and avoiding reverse causality bias. First, the causal effect of severe COVID-19 on each potential mediator (Path A) and of the mediator on PM (Path B) was estimated. The mediation effect was calculated as the product of the two coefficients [β(A) × β(B)]. The proportion mediated was calculated as (mediation effect/total effect) × 100%, with its 95% confidence interval derived using the delta method. A significant mediation effect was declared when all three paths (exposure-mediator, mediator-outcome, and the mediation effect itself) were statistically significant. To account for multiple testing in the mediator screening step, we applied a false discovery rate (FDR) correction using the Benjamini-Hochberg method, with statistical significance defined as FDR-adjusted *p* < 0.05. Detailed procedures for multiple testing correction in the mediation analysis are described in Supplementary material; given the exploratory nature of mediator screening, results should be interpreted as preliminary signals.

### Functional enrichment analysis

Functional enrichment analysis of the mediating plasma proteins (KIAA1024, RNASE1, EGFLAM, CAPZA1, NRG3, IL31) was performed using R software. We first conducted Gene Ontology (GO) Biological Process and Kyoto Encyclopedia of Genes and Genomes (KEGG) pathway enrichment analyses. As no significantly enriched terms survived FDR correction in these two databases, we further performed enrichment analysis using the WikiPathways database. Terms with a FDR < 0.05 were considered statistically significant.

### Clinical cohort and statistical analysis

This clinical analysis served as an ancillary, independent descriptive study accompanying the main genetic investigation. It was designed only to compare admission laboratory parameters between severe and non-severe COVID-19 patients, and did not aim to assess PM incidence, measure the candidate mediators identified by MR, or directly validate MR-inferred causal pathways, and no cases of PM were observed during follow-up.

A single-center retrospective cohort study was conducted to complement the genetic findings. We enrolled 125 consecutive patients with PCR-confirmed COVID-19 admitted to Tianjin Chest Hospital (December 2022 to March 2023). Severe COVID-19 was defined by the presence of respiratory failure. After excluding 17 patients on regular immunosuppressive therapy, 108 patients were analyzed, categorized into severe (*n* = 60) and non-severe (*n* = 48) groups.

Venous blood samples were collected at admission. Hematological parameters, immunoglobulins, complement components, and muscle enzymes were measured using standard automated analyzers. Continuous variables were compared using independent samples *t*-test or Mann–Whitney U test, and categorical variables using the Chi-square test. A two-tailed *p* < 0.05 was considered significant. Clinical analyses were performed using SPSS Statistics (Version 26.0). For multivariable analysis, age- and sex-adjusted logistic regression was performed for each key parameter, with Bonferroni correction for multiple comparisons (corrected *α* = 0.05/8 ≈ 0.00625).

## Results

### Genetic evidence for a potential causal link between COVID-19 and polymyositis

**Figure 2:**
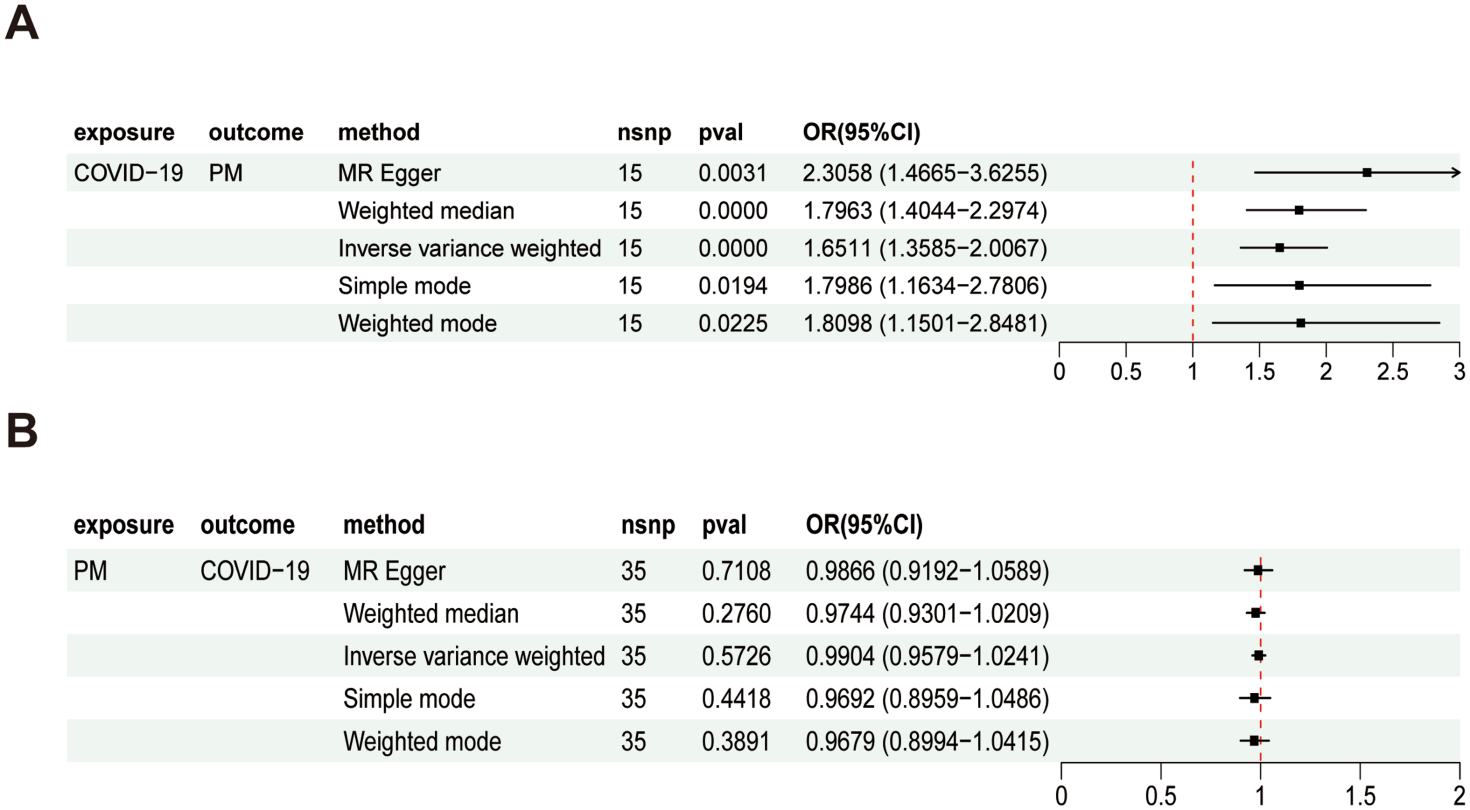
MR analysis of severe COVID-19 and polymyositis (PM): **(A)** Forward MR analysis assessing the causal effect of severe COVID-19 on PM. The results suggest a potential causal effect, with an odds ratio (OR) of 1.65 (95% CI: 1.36–2.01, *p* < 0.01). **(B)** Reverse MR analysis assessing the causal effect of PM on severe COVID-19. The analysis found no evidence of reverse causality (OR: 0.99, 95% CI: 0.96–1.02, *p* > 0.05). Throughout this figure, “PM” refers to polymyositis (the outcome of this study), and “COVID-19” refers to severe COVID-19 (the exposure).

Using SNPs meeting genome-wide significance thresholds for severe COVID-19 (exposure) and PM (outcome), we conducted bidirectional Mendelian randomization (MR) analyses. The results showed that Cochran’s Q test confirmed the absence of heterogeneity, and the MR-Egger intercept indicated no overall pleiotropy. MR analysis provided preliminary genetic evidence consistent with a potential association between COVID-19 infection and polymyositis risk, with an odds ratio (OR) of 1.65 (95% CI: 1.36–2.01, *p* < 0.01) (Figure 2A). No evidence of reverse causality was detected (OR: 0.99, 95% CI: 0.96–1.02, *p* > 0.05) (Figure 2B). Sensitivity analyses supported the robustness of these observations, with Supplementary Figure 1—including scatter plot, leave-one-out analysis, forest plot, and funnel plot—collectively demonstrating the consistency and reliability of the observed association while verifying the absence of significant heterogeneity or pleiotropy.

### Causal effects of plasma multi-omic factors on polymyositis

**Figure 3:**
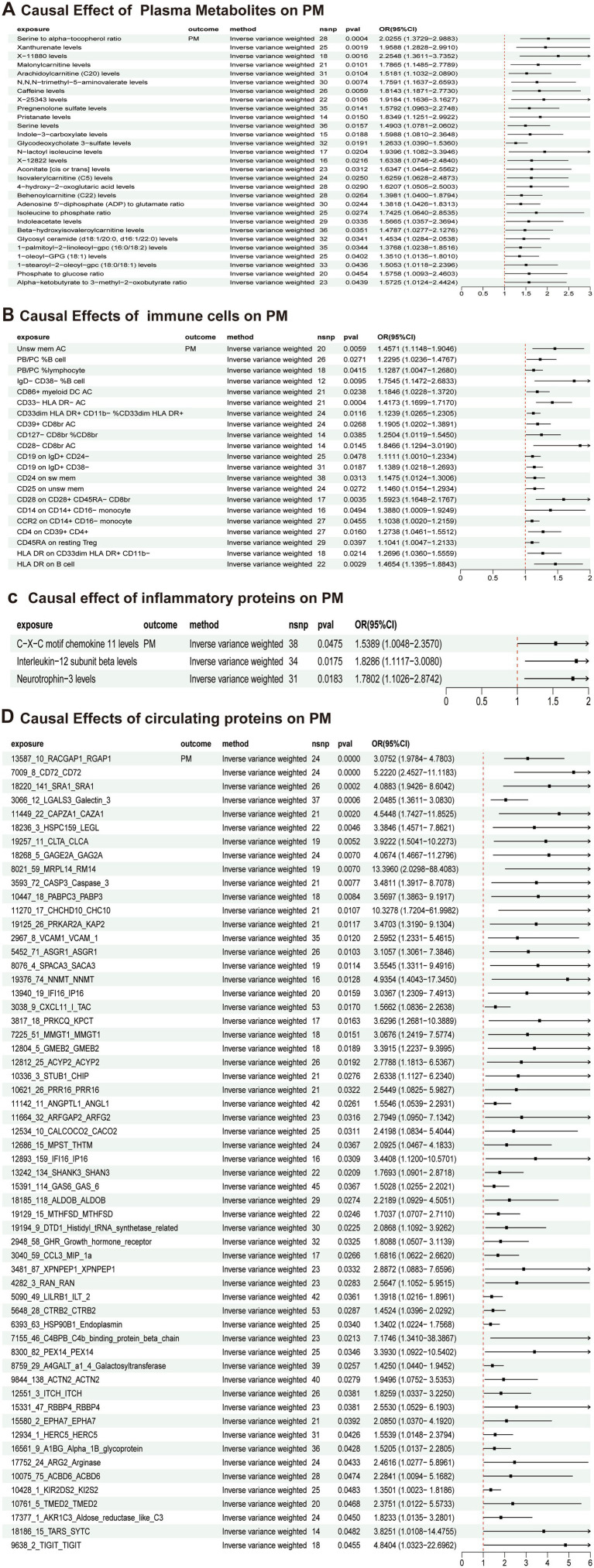
Associations of multi-omic traits with PM risk. **(A)** MR analysis assessing the effects of plasma metabolites on PM. The results suggested 25 plasma metabolites as candidate risk factors. **(B)** MR analysis assessing the effects of immune cell phenotypes on PM. The results suggested 27 immune cell types as candidate risk factors. **(C)** MR analysis assessing the effects of inflammatory proteins on PM. The results suggested three inflammatory proteins as candidate risk factors. **(D)** MR analysis assessing the effects of circulating proteins on PM. The results suggested 63 plasma metabolites as candidate risk factors. Throughout this figure, “PM” refers to polymyositis (the outcome of this study).

Among plasma metabolites, 29 were suggested as risk factors for PM (Figure 3A) while 20 were shown to be protective factors (Supplementary Table 2); immune cell analyses highlighted 21 cell types associated as risk factors (Figure 3B) and 10 as protective (Supplementary Table 3); inflammatory protein analysis indicated three risk factors (Figure 3C) with no protective factors, Circulating protein analysis revealed 58 risk factors (Figure 3D) and 149 protective factors (Supplementary Table 4); and sensitivity analyses supported these findings, demonstrating no significant heterogeneity or horizontal pleiotropy across all MR tests, with all findings adjusted for FDR, detailed methodological supplementary data (including instrument characteristics and F-statistics) for all above analyses available in Supplementary Tables 5–8, and no “convergence” claimed between the clinical and genetic sections as the clinical cohort did not measure the proposed mediators.

### Identification of candidate mediators

To generate hypotheses about potential mediating pathways, we first selected 29 plasma metabolites, 21 immune cell types, 3 inflammatory proteins, and 58 circulating proteins as outcome variables, with severe COVID-19 as the exposure factor. We first screened all the aforementioned factors, retaining only those with a test *p*-value < 0.05; subsequently, we further performed Steiger directionality analysis on the selected eligible factors to clarify the causal direction of the association between these factors and severe COVID-19. This analysis ultimately identified 14 circulating proteins with consistent directional effects, which were then included in the subsequent mediation analysis, where severe COVID-19 was still used as the exposure factor and the 14 selected circulating proteins as the outcome variables (Supplementary Table 9).

**Figure 4:**
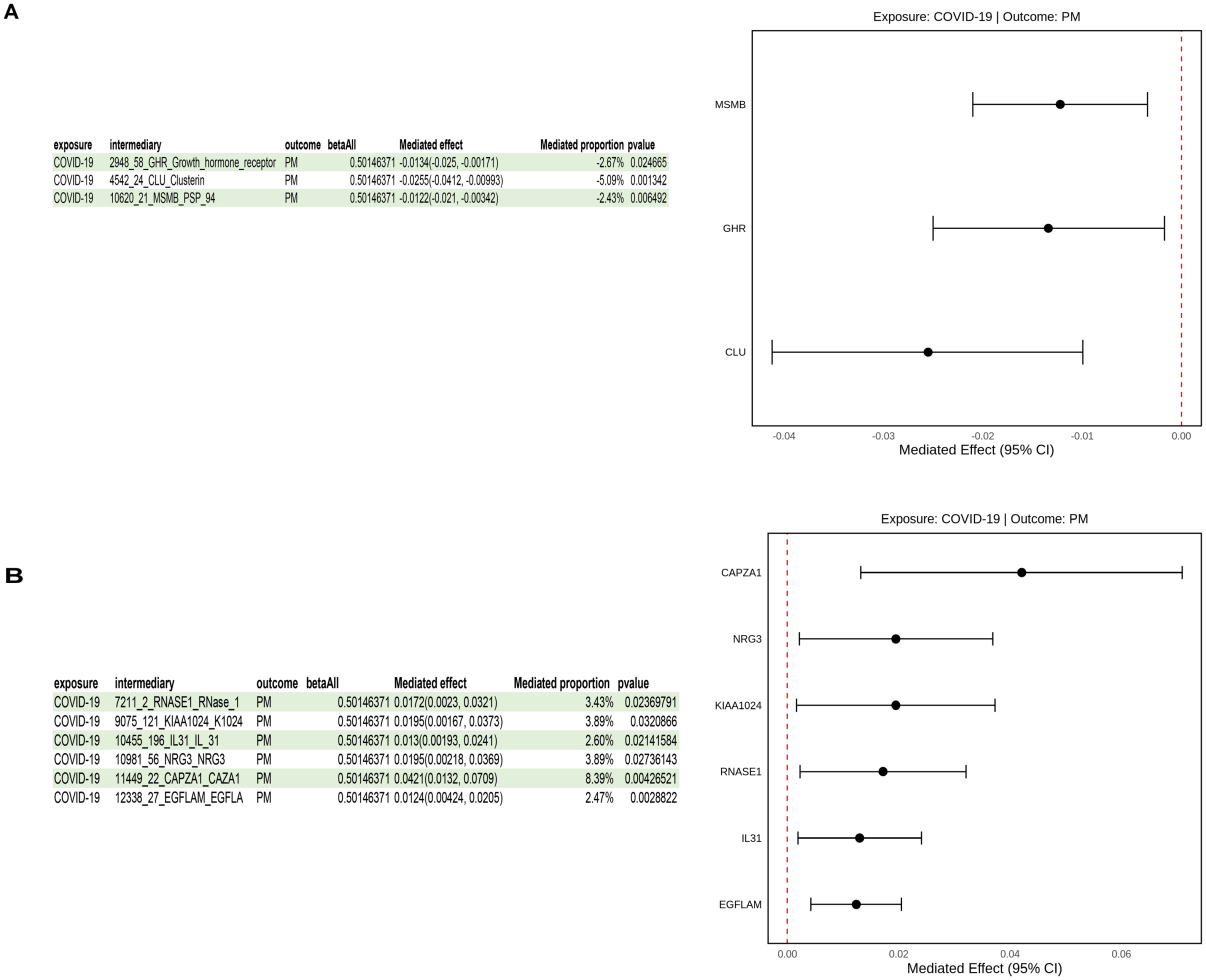
Schematic of potential mediating pathways from severe COVID-19 to PM. **(A)** Analysis identifying the three circulating proteins (GLU, GHR, MSMB) as candidate protect mediators in the pathway. **(B)** Analysis identifying the six circulating proteins (KIAA1024, RNASE1, EGFLAM, CAPZA1, NRG3, IL31) as candidate risk mediators in the pathway. Throughout this table, “PM” refers to polymyositis (the outcome of this study), and “COVID-19” refers to severe COVID-19 (the exposure).

Mediation analyses revealed 9 proteins with consistent directional effects across overall, indirect, and direct estimates: 6 circulating proteins (KIAA1024, RNASE1, EGFLAM, CAPZA1, NRG3, IL31) were identified as potential risk mediators, while 3 were identified as protective mediators (Figure 4). These associations support their potential role as intermediaries in the causal pathway linking severe COVID-19 and PM.

### Enriched pathways of candidate proteins

**Figure 5:**
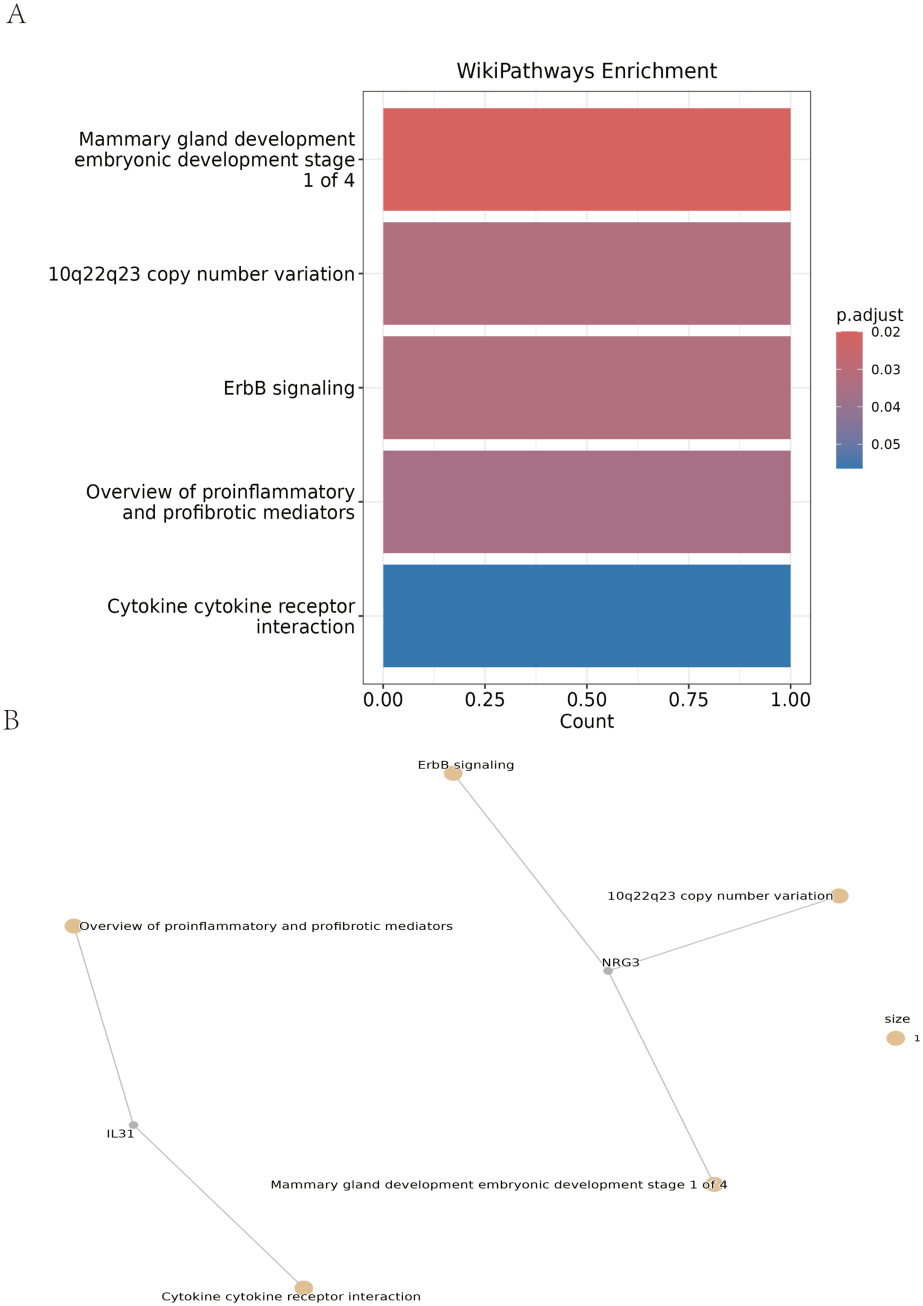
Functional enrichment analysis of the candidate mediating proteins. **(A)** Significantly enriched WikiPathways pathways. **(B)** Interaction network illustrating the functional connections between KIAA1024, RNASE1, EGFLAM, CAPZA1, NRG3, and IL31, highlighting their potential coordinated role.

Functional enrichment analyses were subsequently conducted to explore the biological underpinnings of these candidate mediators. While Gene Ontology (GO) and KEGG pathway analyses did not yield statistically significant results (all *p* > 0.05), WikiPathways enrichment analysis identified several biologically relevant pathways (Figure 5), including mammary gland development (embryonic development stage 1 of 4), 10q22q23 copy number variation, ErbB signaling, and overview of proinflammatory and profibrotic mediators.

Given the lack of statistical significance in GO/KEGG analyses and the exploratory design of the mediation framework, these findings should be interpreted as exploratory signals rather than definitive evidence for causal mediation. Collectively, these results provide preliminary insights into the potential immune and molecular pathways that may mediate the association between severe COVID-19 and the studied outcome.

### Clinical hematological profiles of COVID-19 patients

This study retrospectively included 108 PCR-confirmed COVID-19 patients (48 non-severe cases and 60 severe cases) and analyzed the levels of immune and tissue damage markers at admission (Table 1).

**Table 1:** Comparisons of hematological and immunological indicators between non-severe and severe COVID-19 patients.

Indicators (Unit)	Non-severe group (*n* = 48) (mean ± SD)	Severe group (*n* = 60) (mean ± SD)	*P*-value	Reference range
WBC (×10^9^/L)	9.654 ± 5.252	8.592 ± 4.046	0.116	3.5 ~ 9.5
Neutrophil (×10^9^/L)	6.584 ± 3.769	8.253 ± 5.108	0.007*	1.8 ~ 6.3
Lymphocyte (×10^9^/L)	1.528 ± 2.012	0.802 ± 0.448	0.001*	1.1 ~ 3.2
PLT (×10^9^/L)	263.391 ± 89.779	222.45 ± 82.577	0.001*	125 ~ 350
HB (g/L)	118.805 ± 30.242	124.392 ± 23.753	0.138	Male:130–175; Female:115 ~ 150
IgA (mg/dL)	320.652 ± 159.475	326.4 ± 203.113	0.848	100 ~ 420
IgE (IU/mL)	25.111 ± 13.809	34.896 ± 18.637	0.000*	<165
IgM (mg/dL)	111.737 ± 81.119	98.48 ± 55.885	0.211	30 ~ 220
IgG (mg/dL)	1249.302 ± 405.82	1140.34 ± 372.466	0.077	860 ~ 1740
C3 (mg/dL)	95.089 ± 31.726	87.325 ± 24.754	0.099	70 ~ 140
C4 (mg/dL)	24.265 ± 7.339	24.007 ± 8.186	0.837	10 ~ 40
CK (U/L)	61.458 ± 59.553	328.7 ± 1453.474	0.048*	Male:50 ~ 310; Female:40 ~ 200
CRP (mg/L)	73.041 ± 104.342	95.331 ± 83.279	0.103	≤6
CK-MB (ng/mL)	13.878 ± 5.766	21.288 ± 19.195	0.000*	<5
LDH (U/L)	235.096 ± 66.548	409.75 ± 182.194	0.000*	120 ~ 250
HBDH (U/L)	173.94 ± 51.471	310.36 ± 148.804	0.000*	72 ~ 182

Data were expressed as mean ± standard deviation (SD). Intergroup comparisons were performed using independent samples *t*-test. *P* < 0.05 was considered statistically significant, and statistically significant values were marked with*. WBC, White blood cell count; PLT, Platelet count; HB, Hemoglobin; Ig, Immunoglobulin; C3, Complement 3; C4, Complement 4; CK, Creatine kinase; CRP, C-reactive protein; CK-MB, Creatine kinase-MB; LDH, Lactate dehydrogenase; HBDH, Hydroxybutyrate dehydrogenase.

Compared with non-severe patients, those with severe COVID-19 exhibited significantly lower lymphocyte and platelet counts, higher neutrophil counts, elevated IgE levels, and increased muscle enzymes (CK, CK-MB, LDH, HBDH) (all *p* < 0.05). No significant differences were observed in WBC, hemoglobin, IgG, IgA, IgM, complement C3/C4, or CRP (all *p* > 0.05).

To evaluate independent associations, we performed age- and sex-adjusted logistic regression for each of the eight parameters, with Bonferroni correction for multiple comparisons (corrected *α* = 0.05/8 ≈ 0.00625). As shown in Supplementary Table 10, after adjustment, lymphocyte count (OR = 0.23 per 1 × 10^9^/L, 95% CI: 0.10–0.53, corrected *p* = 0.008), CK-MB (OR = 1.11 per 1 ng/mL, 95% CI: 1.03–1.19, corrected *p* = 0.048), LDH (OR = 1.02 per 1 U/L, 95% CI: 1.01–1.02, corrected *p* < 0.001), and HBDH (OR = 1.02 per 1 U/L, 95% CI: 1.01–1.03, corrected *p* < 0.001) remained significantly associated with severe COVID-19. Neutrophil count, platelet count, IgE, and CK were no longer statistically significant after correction (all corrected *p* > 0.05), suggesting that their univariable associations may be partially explained by confounding by age and sex or by intercorrelations among markers. Detailed results for all tested parameters are provided in Supplementary Table 9.

## Discussion

### Principal findings

This study employed a multilevel approach to investigate the potential link between severe COVID-19 and PM. Our Mendelian randomization (MR) analysis provided genetic evidence consistent with a potential causal effect of severe COVID-19 on increased PM risk (OR = 1.65, 95% CI: 1.36–2.01, *p* < 0.01), with no evidence for reverse causality. Through multi-omic screening and subsequent mediation analysis incorporating Steiger directionality testing, we identified six circulating proteins—KIAA1024, RNASE1, EGFLAM, CAPZA1, NRG3, and IL31—as candidate mediators in the pathway linking severe COVID-19 to PM. WikiPathways enrichment analysis of these candidates revealed several biologically relevant pathways, including ErbB signaling, an overview of proinflammatory and profibrotic mediators, mammary gland development, and 10q22q23 copy number variation pathways. The retrospective clinical profiling was performed independently as a descriptive analysis to characterize the immune and tissue injury features in our cohort. It also provided biological context for genomic inferences. Together, these exploratory findings propose a novel etiological link between severe COVID-19 and PM and identify specific circulating proteins for follow-up investigation.

### Strengths and limitations

A central strength of this study is its multilevel exploratory design, which links genetic epidemiology and clinical observation to investigate a complex hypothesis from complementary perspectives. We applied Mendelian randomization to probe causal link while mitigating confounding. We conducted a systematic multi-omic screen with Steiger directionality testing and FDR correction to produce an unbiased, cross-layer map of candidate mediators.

Several limitations must be considered when interpreting these exploratory results. The genetic data are primarily from European cohorts while the clinical cohort is Chinese, creating an ancestry mismatch that may constrain comparability and generalizability. Although we used the latest FinnGen R12 PM data (287 cases, 484,260 controls), the relatively small number of PM cases may still limit statistical power to detect genetic associations. Our mediation analysis, even with Steiger filtering, remains exploratory; the identified mediators—KIAA1024, RNASE1, EGFLAM, CAPZA1, NRG3, and IL31—replication and functional validation. The clinical component is a single-center, retrospective analysis with a limited sample size (*n* = 108), which limits power and may introduce selection bias. Most importantly, the Mendelian randomization relies on instrumental-variable assumptions; the identified candidates are computational hypotheses requiring prospective and experimental confirmation.

### Interpretation within an integrative hypothesis

While clinical reports have linked COVID-19 and PM (16), the mechanistic remain unclear. We treated the genetic and multi-omic results as a mechanistic hypothesis, and the retrospective clinical analysis serves as an independent descriptive study that characterizes the immune and tissue injury phenotypes of severe COVID-19. This clinical profiling does not provide confirmatory evidence for the causal pathway. Instead, it offers complementary observational support. In the clinical cohort we observed T-cell dysregulation (lymphopenia) and muscle damage (elevated CK, CK-MB, LDH, HBDH) in severe COVID-19. Mediation analysis highlighted enrichment for ErbB signaling, an overview of proinflammatory and profibrotic mediators, mammary gland development, and 10q22q23 copy number variation. Together, these lines of evidence are complementary and hypothesis-generating but do not establish direct causal one-to-one relationships, which require validation in independent cohorts and functional experiments.

ErbB signaling was among the pathway identified by enrichment analysis, with NRG3 as a potential molecular link. NRG3 activates ErbB3/ErbB4 receptors and downstream PI3K/Akt and Ras/MAPK pathways, regulating cell growth, survival, and tissue repair. Studies showed that ErbB receptors can directly bind to the SARS-CoV-2 S1 protein and regulate viral entry and ErbB inhibitors such as lapatinib suppress SARS-CoV-2 and other viruses while protecting against virus-induced inflammation and tissue damage (17). NRG3/ErbB4 signaling can directly drive muscle pathology: in heterotopic ossification models, nerve injury increases NRG3 secretion, activating the PI3K/Akt pathway in fibro-adipogenic progenitors and promoting pathological differentiation (18). In neurodegenerative diseases such as Alzheimer’s disease, NRG/ErbB signaling is closely associated with neuronal survival and repair (19). Together, these findings support a mechanistic link between viral infection, neural function, and muscle integrity, and suggest that NRG3 dysregulation may contribute to post-infectious PM.

The “Overview of proinflammatory and profibrotic mediators” pathway (WikiPathways WP5095) is a curated map of molecules driving tissue inflammation and fibrosis, derived from KEGG COVID-19 disease pathway (ko05171) and literature on prostaglandin-mediated inflammation in COVID-19 (20). It includes a range of cytokines, chemokines, and growth factors—including IL1, IL6, TNF, CCL2, and MMPs—that mediate the transition from acute inflammation to chronic fibrotic remodeling (21). Notably, IL31, one of our identified candidate mediators, is explicitly included in this pathway, supporting its potential role in linking COVID-19 to PM.

RNASE1 (ribonuclease A family member 1) catalyzes RNA hydrolysis. In COVID-19, persistent SARS-CoV-2 RNA in viral reservoirs may drive chronic inflammation and post-acute sequelae. A phase II trial of RSLV-132 (a human RNase1-Fc fusion protein) in PASC patients with severe fatigue showed early improvements in fatigue and physician global assessment, suggesting that enhancing RNase activity to clear persistent viral RNA may reduce post-COVID inflammation (22). Our identification of RNASE1 as a candidate mediator is consistent this model: if persistent viral RNA contributes to chronic inflammation and tissue damage, RNASE1 may act as a counter-regulatory mechanism. Dysregulation of RNASE1 may impair RNA clearance, promote persistent inflammation and increase autoimmune complications such as PM.

F-actin-capping protein subunit alpha 1 (CAPZA1), a member of the capping protein family, is a key regulator of actin filaments (F-actin), that plays an important role in maintaining cytoskeletal dynamic stability. This protein is particularly critical in muscle cells, where it helps maintain the structural and functional integrity of muscle fibers. Dysregulation of CAPZA1 may lead to impaired muscle development and dysfunction, although the underlying mechanisms require further investigation.

EGFLAM (pikachurin) is an extracellular matrix-like protein first described in retinal neurobiology, where it plays a critical role in the development and maintenance of photoreceptor ribbon synapses by mediating synaptic structural integrity through interactions with the dystrophin-glycoprotein complex (23). In congenital muscular dystrophy, EGFLAM deficiency disrupts its interaction with the dystroglycan complex, directly contributing to the pathogenesis of muscle-eye-brain disease (24). Our study identifies EGFLAM as a candidate mediator, suggesting that infection may compromise extracellular matrix (ECM) integrity via EGFLAM dysregulation, leading to chronic tissue remodeling and increased susceptibility to autoimmunity.

IL-31, a cytokine belonging to the IL-6 family, was identified as a candidate mediator. As a proinflammatory cytokine, IL-31 is implicated in various diseases, including atopic dermatitis, chronic pruritus, and autoimmune conditions such as systemic lupus erythematosus and rheumatoid arthritis (25, 26). In the context of COVID-19, IL-31 may contribute to the hyperinflammatory response, and its upregulation could promote chronic inflammation that predisposes to autoimmune complications like PM.

As a separate descriptive background analysis, we retrospectively characterized the hematological and biochemical profiles of COVID-19 patients at admission. The primary aim of this analysis was to define the clinical phenotype of severe COVID-19 in our study cohort, thereby providing biological context for the immune and metabolic pathways inferred from genomic data. The study identified significant immune cell imbalances—including lymphocytopenia (linked to T-cell exhaustion and secondary infection risk) (5) neutrophilia (indicative of excessive inflammation, lung injury, and thrombosis) (27), and thrombocytopenia (reflecting endothelial damage or coagulopathy and predicting poor prognosis) (28)—which collectively contribute to disease progression. Concurrently, elevated markers of multi-organ damage were observed: increased serum CK/CK-MB suggesting potential myocardial or skeletal muscle injury due to viral invasion or hypoxic stress (29) and significantly raised LDH/HBDH levels reflecting broad cellular necrosis and correlating with mortality (30). Notably, humoral immunity markers (IgG, IgM, IgA, C3, C4) and CRP showed no significant association with severity. After adjusting for age and sex, lymphocyte count, CK-MB, LDH, and HBDH remained independently associated with severe COVID-19. The persistence of LDH and HBDH signals underscores the role of tissue injury in disease progression, while the loss of significance for CK and platelet count may reflect confounding or limited power. This lack of association—potentially attributable to the early stage of admission, comparable complement consumption across groups, or the immunosuppressive state in severe cases—suggests that the early progression to severe COVID-19 is less dependent on canonical antibody and complement pathways.

Notably, this clinical profile is characterized by lymphocytopenia (reflecting T-cell depletion) and elevated LDH (suggesting muscle injury). These phenotypic findings support the biological pathways implicated by our genetic studies, including ErbB signaling dysregulation (via NRG3), proinflammatory and profibrotic processes (via RNASE1, CAPZA1, EGFLAM), and IL-31-mediated inflammation in severe COVID-19. While these findings offer mechanistic context, they do not represent formal validation of the genetically inferred causal relationships.

### Synthesis and implications

Collectively, these multi-omic findings converge into a testable hypothesis: Severe SARS-CoV-2 infection may disrupt ErbB signaling, while activating proinflammatory and profibrotic mediators (mediated by RNASE1, CAPZA1, and EGFLAM), and IL-31-drivened inflammation. Given COVID-19’s persistent global burden, our findings reveal its potential to trigger autoimmune responses. By integrating multi-omic clues, we have identified specific circulating proteins that coalesce into a multifactorial framework linking COVID-19 to PM. This study provides a foundational hypothesis for further exploration and prioritizes avenues, particularly around ErbB signaling (via NRG3), RNA clearance mechanisms (via RNASE1), cytoskeletal regulation (via CAPZA1), matrix remodeling (via EGFLAM), and IL-31-mediated inflammation, for future experimental and therapeutic investigation.

## Conclusion

In conclusion, this multilevel study provides genetic evidence supporting a potential causal link between COVID-19 susceptibility and polymyositis, identifying six circulating proteins—KIAA1024, RNASE1, EGFLAM, CAPZA1, NRG3, and IL31—as candidate mediators. WikiPathways enrichment analysis implicates ErbB signaling, Overview of proinflammatory and profibrotic mediators, mammary gland development, and 10q22q23 copy number variation as key contributors. Retrospective clinical profiling conducted as an independent observational analysis, revealed T-cell depletion and tissue injury patterns in severe COVID-19 that parallel the genetically inferred pathways—though this descriptive alignment does not establish causation.

Future research should focus on experimental validation of these candidates, particularly NRG3 (ErbB signaling), RNASE1 (RNA clearance), CAPZA1 (cytoskeletal regulation), EGFLAM (matrix remodeling), and IL31 (proinflammatory signaling). Prospective Studies should explore their biomarker potential in post-COVID cohorts and evaluate whether therapeutic targeting these pathways (ErbB signaling, RNA clearance, cytoskeletal regulation, matrix remodeling, or IL-31 signaling) could mitigate disease progression. Additionally, clinical signatures (e.g., persistent lymphopenia, elevated LDH/CK-MB without other causes) should be investigated as predictors of PM in COVID-19 convalescents.

## Data Availability

The summary-level GWAS data used in this study are publicly available from the IEU GWAS database (https://gwas.mrcieu.ac.uk/). The raw clinical data of the COVID-19 cohort are available from the corresponding authors upon reasonable request, with approval from the Ethics Committee of Tianjin Chest Hospital. All R code for MR analysis and functional enrichment analysis is available from the corresponding authors upon reasonable request.
