# Supplementary figures and images for: A descriptive multilevel analysis associating COVID-19 with polymyositis: from genetic markers and candidate mediators to clinical hematological profiles
Source: Front Med (Lausanne). 2026 Apr 9;13:1775960. doi: 10.3389/fmed.2026.1775960 (PMC13102858; doi:10.3389/fmed.2026.1775960)

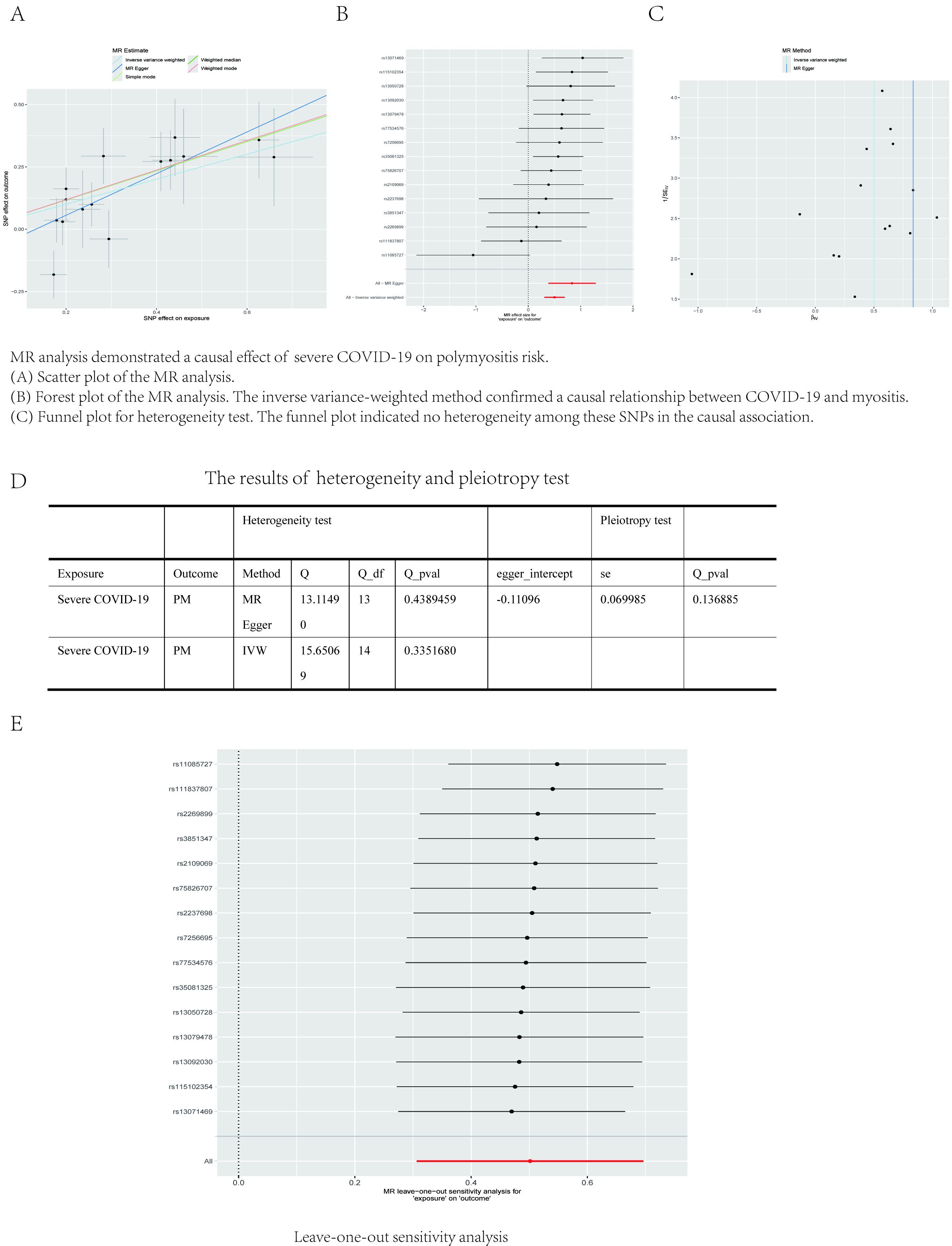

Supplement: Supplementary file 1 [file Image_1.tif]
