# Supplementary material for: A descriptive multilevel analysis associating COVID-19 with polymyositis: from genetic markers and candidate mediators to clinical hematological profiles
Source: Front Med (Lausanne). 2026 Apr 9;13:1775960. doi: 10.3389/fmed.2026.1775960 (PMC13102858; doi:10.3389/fmed.2026.1775960)
Supplement: Supplementary file 3 [file Supplementary_file_2.docx]

**STROBE-MR checklist of recommended items to address in reports of Mendelian randomization studies**^1^ ^2^

| **Item No.** | **Section** | **Checklist item** | **Page No.** | **Relevant text from manuscript** |
| --- | --- | --- | --- | --- |
| 1 | **TITLE and ABSTRACT** | Indicate Mendelian randomization (MR) as the study’s design in the title and/or the abstract if that is a main purpose of the study | 1 | Title: A Descriptive Multilevel Analysis Associating COVID-19 with Polymyositis: From Genetic Markers and Candidate Mediators to Clinical Hematological Profiles; Abstract: This study adopted a multilevel exploratory framework to investigate potential relationships: we used two-sample Mendelian randomization (MR) to evaluate genetic evidence for a potential association between severe COVID-19 and PM, conducted exploratory multi-omic mediator screening, and performed a retrospective clinical analysis to characterize hematological profiles in patients with severe COVID-19 |
|  | **INTRODUCTION** |  |  |  |
| 2 | **Background** | Explain the scientific background and rationale for the reported study. What is the exposure? Is a potential causal relationship between exposure and outcome plausible? Justify why MR is a helpful method to address the study question | 2 | Background: COVID-19 pandemic has autoimmune sequelae including polymyositis (PM), observational studies suggest an association but are confounded by reverse causality and confounding; Exposure: Severe COVID-19; Outcome: Polymyositis (PM); Plausibility: COVID-19 induces immune dysregulation, inflammatory cytokine elevation, metabolic abnormalities and plasma protein profile alterations that overlap with PM pathogenesis, and viral myositis after COVID-19 is documented; MR Rationale: MR uses genetic variants as instrumental variables to assess potential causal relationships while minimizing confounding, addressing the limitations of observational studies for this research question. |
| 3 | **Objectives** | State specific objectives clearly, including pre-specified causal hypotheses (if any). State that MR is a method that, under specific assumptions, intends to estimate causal effects | 2-3 | Objectives: 1) Use two-sample MR to investigate if genetic predisposition to severe COVID-19 is associated with increased PM risk (pre-specified causal hypothesis: severe COVID-19 has a causal effect on elevated PM risk); 2) Implement an exploratory multi-omic MR framework to screen candidate mediating biomarkers across inflammatory proteins, immune cell phenotypes, metabolites and circulating plasma proteins; 3) Perform a retrospective clinical analysis to characterize hematological and muscle enzyme profiles in severe vs. non-severe COVID-19 patients to provide biological context for genetic findings; MR Statement: Mendelian randomization (MR) is a genetic epidemiological method that uses genetic variants as instrumental variables (IVs) to assess evidence for potential causal relationships while minimizing confounding, with causal effect estimation reliant on the satisfaction of key IV assumptions (relevance, independence, no horizontal pleiotropy). |
|  | **METHODS** |  |  |  |
| 4 | **Study design and data sources** | Present key elements of the study design early in the article. Consider including a table listing sources of data for all phases of the study. For each data source contributing to the analysis, describe the following: |  |  |
|  | a) | Setting: Describe the study design and the underlying population, if possible. Describe the setting, locations, and relevant dates, including periods of recruitment, exposure, follow-up, and data collection, when available. | 3-4 | Setting: MR analysis - public summary-level GWAS data with European ancestry (consistent across exposure, outcome, mediators) from international consortia/initiatives; Clinical analysis - Tianjin Chest Hospital, China, December 2022 to March 2023 (recruitment and data collection); |
|  | b) | Participants: Give the eligibility criteria, and the sources and methods of selection of participants. Report the sample size, and whether any power or sample size calculations were carried out prior to the main analysis | 3-4 | Participants: MR - Severe COVID-19 (COVID-19 Host Genetics Initiative, release 5, GCST011075: 5,101 cases, 1,383,241 controls); PM (FinnGen consortium, release R12: 287 cases, 484,260 controls); Mediators (91 inflammatory proteins: 14,824 individuals; 731 immunocyte phenotypes: 3,757 Sardinians; 1,400 metabolites: 8,299 samples; 4,907 plasma proteins: 35,559 European ancestry individuals); no pre-specified power/sample size calculation for MR; Clinical - 125 consecutive PCR-confirmed COVID-19 inpatients, 17 excluded (regular immunosuppressive therapy), 108 analyzed (60 severe, 48 non-severe); eligibility: PCR-confirmed COVID-19 admission, exclusion: regular immunosuppressive therapy; |
|  | c) | Describe measurement, quality control and selection of genetic variants | 3-4 | Genetic variants: GWAS harmonization via TwoSampleMR R package (v0.5.6) (strand alignment, allele frequency matching); palindromic SNPs harmonized with harmonise_data (action=2), ambiguous SNPs excluded; IV selection with P < 5×10⁻⁸ (genome-wide significant) or P < 1×10⁻⁵ (relaxed) threshold, LD clumping (r² < 0.001, 10,000 kb window, 1000 Genomes European reference); SNPs associated with PM (P < 0.05) excluded; F-statistic >10 for instrument strength; MR-PRESSO test to exclude horizontal pleiotropy outliers; 15 SNPs retained for primary MR; |
|  | d) | For each exposure, outcome, and other relevant variables, describe methods of assessment and diagnostic criteria for diseases | 3 | Exposure/Outcome/Variables: Severe COVID-19 (defined by COVID-19 Host Genetics Initiative); PM (defined by FinnGen consortium); Severe COVID-19 (clinical) - defined by presence of respiratory failure; Hematological/immunological/muscle enzyme variables - measured via standard automated analyzers at admission; |
|  | e) | Provide details of ethics committee approval and participant informed consent, if relevant | 5 | Ethics: Clinical study - approved by Ethics Committee of Tianjin Chest Hospital (2025LW-031), informed consent waived (retrospective), data anonymized; GWAS data - original studies obtained ethical approval and participant consent. |
| 5 | **Assumptions** | Explicitly state the three core IV assumptions for the main analysis (relevance, independence and exclusion restriction) as well assumptions for any additional or sensitivity analysis | 3 | Core IV Assumptions: 1) Relevance: Genetic variants are robustly associated with the exposure (severe COVID-19); 2) Independence: Genetic variants are not associated with confounding factors of the severe COVID-19-PM relationship; 3) Exclusion restriction: Genetic variants influence PM only through their effect on severe COVID-19 (no horizontal pleiotropy); Additional/Sensitivity Analysis Assumptions: For bidirectional MR - the same core IV assumptions apply to the reverse direction (PM as exposure, severe COVID-19 as outcome); For mediation analysis - causal direction (severe COVID-19→mediator→PM) is consistent with research hypothesis (no reverse causality between exposure-mediator and mediator-outcome). |
| 6 | **Statistical methods: main analysis** | Describe statistical methods and statistics used |  |  |
|  | a) | Describe how quantitative variables were handled in the analyses (i.e., scale, units, model) | 3-5 | Quantitative variables (clinical): Continuous variables compared via independent samples t-test/Mann-Whitney U test; multivariable age- and sex-adjusted logistic regression; MR quantitative variables: effect sizes reported as odds ratios (OR) with 95% confidence intervals (CI); |
|  | b) | Describe how genetic variants were handled in the analyses and, if applicable, how their weights were selected | 4 | Genetic variants: Harmonized (strand/allele frequency), clumped for independence, filtered for strength (F>10) and no direct outcome association; weighted via inverse-variance weighting (IVW) for primary MR analysis; |
|  | c) | Describe the MR estimator (e.g. two-stage least squares, Wald ratio) and related statistics. Detail the included covariates and, in case of two-sample MR, whether the same covariate set was used for adjustment in the two samples | 4 | MR Estimator: Primary - inverse-variance weighted (IVW); supplementary - weighted median, MR-Egger, weighted mode; Two-sample MR - no additional covariates used (GWAS data already adjusted for population stratification/ancestry, consistent European ancestry across exposure/outcome/mediator samples); |
|  | d) | Explain how missing data were addressed | 3 | Missing data: Subsequently, one SNP (rs2597569) was identified as a horizontal pleiotropy outlier by the MR-PRESSO test and was excluded, leaving 15 SNPs for the primary MR analysis (Supplementary Table S1); no missing data reported for clinical quantitative variables (standard automated analyzer measurements); |
|  | e) | If applicable, indicate how multiple testing was addressed | 4，5 | we applied a false discovery rate (FDR) correction using the Benjamini-Hochberg method, with statistical significance defined as FDR-adjusted P < 0.05. For multivariable analysis, age- and sex-adjusted logistic regression was performed for each key parameter, with Bonferroni correction for multiple comparisons (corrected α = 0.05/8 ≈ 0.00625). |
| 7 | **Assessment of assumptions** | Describe any methods or prior knowledge used to assess the assumptions or justify their validity | 4 | Assumption Assessment: 1) Relevance - F-statistic >10 for all IVs to confirm instrumental variable strength; 2) Independence - LD clumping to ensure genetic variant independence, exclusion of variants associated with PM (P < 0.05) to avoid confounding; 3) No horizontal pleiotropy - MR-Egger intercept test, MR-PRESSO global test, Cochran’s Q test for heterogeneity; 4) Causal direction (mediation) - Steiger directionality tests to confirm severe COVID-19→mediator and mediator→PM; Prior Knowledge Justification: Consistent European ancestry across all GWAS datasets to avoid population stratification (a key confounder); use of well-validated public GWAS datasets from established consortia/initiatives with rigorous quality control in original studies. |
| 8 | **Sensitivity analyses and additional analyses** | Describe any sensitivity analyses or additional analyses performed (e.g. comparison of effect estimates from different approaches, independent replication, bias analytic techniques, validation of instruments, simulations) | 4，5 | Sensitivity Analyses: MR - Cochran’s Q test (heterogeneity); MR-Egger intercept, MR-PRESSO global test (horizontal pleiotropy); leave-one-out analysis (individual variant influence); supplementary MR estimators (weighted median, MR-Egger, weighted mode) for robustness; Bidirectional MR (to assess reverse causality: PM→severe COVID-19); Additional Analyses: Multi-omic MR screening (metabolites, immune cells, inflammatory proteins, plasma proteins) for PM risk factors; Two-step MR mediation analysis (to identify candidate mediators); Functional enrichment analysis (GO, KEGG, WikiPathways) of candidate mediators; Clinical - univariable comparison of ematological/immunological/muscle enzyme indices; age/sex-adjusted multivariable logistic regression for severe COVID-19 association. |
| 9 | **Software and pre-registration** |  |  |  |
|  | a) | Name statistical software and package(s), including version and settings used | 3，5 | Software: MR/Functional enrichment - R software (TwoSampleMR v0.5.6); Clinical - SPSS Statistics (Version 26.0) |
|  | b) | State whether the study protocol and details were pre-registered (as well as when and where) | - | Pre-registration: No study protocol or details were pre-registered |
|  | **RESULTS** |  |  |  |
| 10 | **Descriptive data** |  |  |  |
|  | a) | Report the numbers of individuals at each stage of included studies and reasons for exclusion. Consider use of a flow diagram | 3，4 | MR - Severe COVID-19: 5,101 cases/1,383,241 controls (no exclusion); PM:287 cases/484,260 controls (no exclusion); Genetic variants: initial harmonized SNPs → 1 excluded (MR-PRESSO pleiotropy outlier) →15 retained for primary MR; Clinical - 125 consecutive COVID-19 inpatients →17 excluded (regular immunosuppressive therapy) →108 analyzed (60 severe, 48 non-severe); Study workflow flow diagram provided (Figure 1) |
|  | b) | Report summary statistics for phenotypic exposure(s), outcome(s), and other relevant variables (e.g. means, SDs, proportions) | 5，6 | Clinical summary statistics: Mean ± SD for all hematological/immunological/muscle enzyme indices in severe/non-severe COVID-19 groups (Table 1); MR: OR (95% CI) for genetic variant associations with exposure/outcome/mediators; |
|  | c) | If the data sources include meta-analyses of previous studies, provide the assessments of heterogeneity across these studies | - | No meta-analyses of previous studies used for data sources |
|  | d) | For two-sample MR:  i.  Provide justification of the similarity of the genetic variant-exposure associations between the exposure and outcome samples  ii.  Provide information on the number of individuals who overlap between the exposure and outcome studies | 3 | Two-sample MR: i. Justification - consistent European ancestry across all exposure/outcome/mediator GWAS datasets, GWAS harmonization (strand alignment, allele frequency matching) to ensure consistent genetic variant-exposure associations; ii. Overlap - no information on individual participant overlap between exposure/outcome GWAS studies (summary-level GWAS data with no individual level identifiers). |
| 11 | **Main results** |  |  |  |
|  | a) | Report the associations between genetic variant and exposure, and between genetic variant and outcome, preferably on an interpretable scale | 3 | Genetic variant-exposure/outcome associations: Reported as OR (95% CI) with P-values for all multi-omic traits (metabolites, immune cells, proteins) with PM (Figure 3, Supplementary Tables); 15 retained SNPs show robust association with severe COVID-19 (F>10, Supplementary Table S1); |
|  | b) | Report MR estimates of the relationship between exposure and outcome, and the measures of uncertainty from the MR analysis, on an interpretable scale, such as odds ratio or relative risk per SD difference | 5 | MR Estimate (severe COVID-19→PM): IVW OR=1.65 (95% CI:1.36-2.01, P<0.01); no reverse causality (PM→severe COVID-19: OR=0.99, 95% CI:0.96-1.02, P>0.05) |
|  | c) | If relevant, consider translating estimates of relative risk into absolute risk for a meaningful time period | - | Absolute risk translation not performed (exploratory study with no longitudinal follow-up for PM incidence |
|  | d) | Consider plots to visualize results (e.g. forest plot, scatterplot of associations between genetic variants and outcome versus between genetic variants and exposure) | 13，14，15，16，17 | Visualization plots: MR analysis forest plots (Figure 2); multi-omic trait-PM association plots (Figure 3); mediation effect plots (Figure 4); functional enrichment analysis plots (Figure 5); leave-one-out/scatter/funnel plots for MR sensitivity (Supplementary Figure 1). |
| 12 | **Assessment of assumptions** |  |  |  |
|  | a) | Report the assessment of the validity of the assumptions | 3 | Validity Assessment: Relevance - all IVs with F-statistic >10 (Supplementary Tables); Independence - no genetic variants associated with PM (P<0.05) included; No horizontal pleiotropy - MR-Egger intercept indicated no overall pleiotropy, MR-PRESSO test identified 1 outlier (excluded), Cochran’s Q test confirmed no heterogeneity; Causal direction - Steiger directionality tests confirmed severe COVID-19→mediator→PM for candidate mediators |
|  | b) | Report any additional statistics (e.g., assessments of heterogeneity across genetic variants, such as *I^2^*, Q statistic or E-value) | Supplementary Figure 1 | Heterogeneity Statistics: Cochran’s Q test (no heterogeneity for primary MR analysis, P>0.05); no I2/E-value reported (no significant heterogeneity detected). |
| 13 | **Sensitivity analyses and additional analyses** |  |  |  |
|  | a) | Report any sensitivity analyses to assess the robustness of the main results to violations of the assumptions | 5-6 | Robustness Sensitivity: Supplementary MR estimators (weighted median, MR-Egger, weighted mode) consistent with IVW results; leave-one-out analysis confirmed no single variant drives the severe COVID-19→PM association; FDR correction for mediation analysis confirmed 6 circulating proteins as candidate risk mediators |
|  | b) | Report results from other sensitivity analyses or additional analyses | 5-7 | Additional Analyses: Multi-omic MR identified 29 metabolite,21 immune cell,3 inflammatory protein,58 plasma protein risk factors for PM; Mediation analysis identified 6 risk mediators (KIAA1024,RNASE1,EGFLAM,CAPZA1,NRG3,IL31) and 3 protective mediators for severe COVID-19→PM; WikiPathways enrichment identified proinflammatory/profibrotic mediators and ErbB signaling as key pathways; Clinical analysis identified lymphopenia,neutrophilia,thrombocytopenia,elevated IgE/muscle enzymes in severe COVID-19 |
|  | c) | Report any assessment of direction of causal relationship (e.g., bidirectional MR) | 5-7 | Causal Direction: Bidirectional MR confirmed no reverse causality (PM→severe COVID-19: OR=0.99,95%CI:0.96-1.02,P>0.05); Steiger tests confirmed exposure-mediator-outcome direction |
|  | d) | When relevant, report and compare with estimates from non-MR analyses | - | Non-MR Comparison: No direct comparison (clinical analysis is independent descriptive study, not designed to test MR hypotheses); clinical findings (lymphopenia, muscle damage) align with MR-inferred biological pathways; |
|  | e) | Consider additional plots to visualize results (e.g., leave-one-out analyses) | 16-17 | Additional Plots: Leave-one-out/scatter/funnel plots (Supplementary Figure 1); mediation effect forest plots (Figure 4); functional enrichment network plots (Figure 5). |
|  | **DISCUSSION** |  |  |  |
| 14 | **Key results** | Summarize key results with reference to study objectives | 5-7 | Key Results (aligned with objectives): 1) MR analysis provided genetic evidence for a potential causal effect of severe COVID-19 on increased PM risk (IVW OR=1.65,95%CI:1.36-2.01,P<0.01) with no reverse causality; 2) Multi-omic MR mediation analysis identified 6 circulating proteins (KIAA1024,RNASE1,EGFLAM,CAPZA1,NRG3,IL31) as candidate risk mediators, enriched in proinflammatory/profibrotic and ErbB signaling pathways; 3) Retrospective clinical analysis characterized severe COVID-19 with distinct hematological profiles (lymphopenia, neutrophilia, thrombocytopenia, elevated IgE/CK-MB/LDH/HBDH), with lymphocyte count, CK-MB, LDH, HBDH independently associated with severe COVID-19 after age/sex adjustment; clinical phenotypes align with MR-inferred biological pathways, providing phenotypic plausibility. |
| 15 | **Limitations** | Discuss limitations of the study, taking into account the validity of the IV assumptions, other sources of potential bias, and imprecision. Discuss both direction and magnitude of any potential bias and any efforts to address them | 8 | Limitations & Bias: 1) IV Assumptions - MR findings rely on IV validity; while pleiotropy was tested (MR-Egger/MR-PRESSO), unmeasured pleiotropy cannot be fully excluded; 2) Population Mismatch - MR data (European ancestry) vs. clinical cohort (Chinese descent) limits generalizability and comparability; 3) PM Sample Size - FinnGen PM data (287 cases) has modest statistical power for genetic association; addressed via F-statistic >10 to minimize weak instrument bias; 4) Mediation Analysis - exploratory in design, candidate mediators require independent validation; addressed via Steiger directionality tests and FDR correction; 5) Clinical Study - single-center, retrospective, small sample (n=108) with selection bias and limited power; no PM cases observed in follow-up; 6) No Convergence - clinical cohort did not measure MR-identified mediators, no direct validation of causal pathways; 7) Imprecision - functional enrichment analysis had no significant GO/KEGG results, only WikiPathways enrichment (exploratory signals); Efforts to Address: Rigorous IV selection, multiple sensitivity analyses, FDR/Bonferroni correction for multiple testing, data anonymization for clinical study, use of well-validated public GWAS datasets. |
| 16 | **Interpretation** |  |  |  |
|  | a) | Meaning: Give a cautious overall interpretation of results in the context of their limitations and in comparison with other studies | 8-11 | Meaning: This exploratory multilevel study provides preliminary genetic clues (not definitive evidence) for a potential severe COVID-19-PM causal link, consistent with clinical case reports of post-COVID-19 PM; candidate mediators and clinical phenotypes provide biological plausibility, but findings require independent validation due to study limitations |
|  | b) | Mechanism: Discuss underlying biological mechanisms that could drive a potential causal relationship between the investigated exposure and the outcome, and whether the gene-environment equivalence assumption is reasonable. Use causal language carefully, clarifying that IV estimates may provide causal effects only under certain assumptions | 8-10 | Mechanism: Severe COVID-19 may disrupt ErbB signaling (via NRG3), impair RNA clearance (via RNASE1), alter cytoskeletal/matrix integrity (via CAPZA1/EGFLAM), and drive proinflammatory signaling (via IL31) → create a dysregulated proinflammatory environment → lower threshold for autoimmune muscle targeting (PM); Gene-Environment Equivalence: Reasonable for exploratory purposes (severe COVID-19 genetic predisposition mimics environmental exposure to severe COVID-19), but unproven for this specific association; Causal Language: Emphasized that MR results are preliminary genetic evidence for a potential causal effect, only valid under IV assumptions, and findings are exploratory clues not definitive mechanistic evidence; |
|  | c) | Clinical relevance: Discuss whether the results have clinical or public policy relevance, and to what extent they inform effect sizes of possible interventions | 10-11 | Clinical/Public Policy Relevance: Identifies candidate proteins (NRG3,RNASE1,IL31) and pathways (ErbB, proinflammatory/profibrotic) as potential therapeutic targets for post-COVID-19 autoimmune complications (including PM); Clinical signatures (persistent lymphopenia, elevated LDH/CK-MB) may be potential predictive biomarkers for PM risk in convalescent COVID-19 patients; Informs public health awareness of long-term autoimmune sequelae of severe COVID-19; Effect Sizes: MR OR=1.65 provides a preliminary effect size estimate, but no clinical intervention effect sizes can be inferred (requires prospective experimental studies). |
| 17 | **Generalizability** | Discuss the generalizability of the study results (a) to other populations, (b) across other exposure periods/timings, and (c) across other levels of exposure | 7-11 | a) Other Populations: Limited generalizability to non-European/non-Chinese populations due to MR (European) and clinical (Chinese) ancestry mismatch; no data for pediatric/geriatric subgroups or patients with comorbidities; b) Exposure Periods/Timings: MR assesses genetic predisposition to severe COVID-19 (lifetime exposure), not acute COVID-19 infection timing (acute/post-acute); clinical data from December 2022-March 2023 (specific COVID-19 wave) limits generalizability to other pandemic periods; c) Other Exposure Levels: MR focused on severe COVID-19 only; no data for mild/moderate COVID-19 and PM risk; clinical study only compared severe vs. non-severe COVID-19, no gradient analysis of disease severity and PM-related phenotypes. |
|  | **OTHER INFORMATION** |  |  |  |
| 18 | **Funding** | Describe sources of funding and the role of funders in the present study and, if applicable, sources of funding for the databases and original study or studies on which the present study is based | 11 | Funding for Present Study: National Clinical Key Specialty Construction Project; Tianjin Key Medical Discipline Project (TJYXZDXK-3-032C); Tianjin Health Science and Technology Clinical Key Specialty Project (TJWJ2024ZK003) |
| 19 | **Data and data sharing** | Provide the data used to perform all analyses or report where and how the data can be accessed, and reference these sources in the article. Provide the statistical code needed to reproduce the results in the article, or report whether the code is publicly accessible and if so, where | 5 | The summary-level GWAS data used in this study are publicly available from the IEU GWAS database (https://gwas.mrcieu.ac.uk/). The raw clinical data of the COVID-19 cohort are available from the corresponding authors upon reasonable request, with approval from the Ethics Committee of Tianjin Chest Hospital. All R code for MR analysis and functional enrichment analysis is available from the corresponding authors upon reasonable request |
| 20 | **Conflicts of Interest** | All authors should declare all potential conflicts of interest | 11 | The authors declare that the research was conducted in the absence of any commercial or financial relationships that could be construed as a potential conflict of interest. |

This checklist is copyrighted by the Equator Network under the Creative Commons Attribution 3.0 Unported (CC BY 3.0) license.

1. Skrivankova VW, Richmond RC, Woolf BAR, Yarmolinsky J, Davies NM, Swanson SA, et al. Strengthening the Reporting of Observational Studies in Epidemiology using Mendelian Randomization (STROBE-MR) Statement. JAMA. 2021;under review.

2. Skrivankova VW, Richmond RC, Woolf BAR, Davies NM, Swanson SA, VanderWeele TJ, et al. Strengthening the Reporting of Observational Studies in Epidemiology using Mendelian Randomisation (STROBE-MR): Explanation and Elaboration. BMJ. 2021;375:n2233.
